# Health state utilities associated with attributes of weekly injection devices for treatment of type 2 diabetes

**DOI:** 10.1186/s12913-017-2648-7

**Published:** 2017-11-25

**Authors:** Louis S. Matza, Kristina S. Boye, Katie D. Stewart, Evan W. Davies, Rosirene Paczkowski

**Affiliations:** 1 Outcomes Research, Evidera, 7101 Wisconsin Avenue, Suite 1400, Bethesda, MD 20814 USA; 2 Eli Lilly and Company, Indianapolis, IN USA; 3 Formerly with Outcomes Research, Evidera, London, UK

**Keywords:** Utility, type 2 diabetes, Injection process, GLP-1 receptor agonist, Time trade-off, Weekly injection

## Abstract

**Background:**

Glucagon-like peptide-1 (GLP-1) receptor agonists are often recommended as part of combination therapy for type 2 diabetes when oral medication does not result in sufficient glycemic control. Several GLP-1 receptor agonists are available as weekly injections. These medications vary in their injection delivery systems, and these differences could impact quality of life and treatment preference. The purpose of this study was to estimate utilities associated with attributes of injection delivery systems for weekly GLP-1 therapies.

**Methods:**

Participants with type 2 diabetes in the UK valued health states in time trade-off interviews. The health states (drafted based on literature, device instructions for use, and clinician interviews) had identical descriptions of type 2 diabetes, but differed in description of the treatment process. One health state described oral treatment, while six others described oral treatment plus a weekly injection. The injection health states varied in three aspects of the treatment administration process: requirements for reconstituting the medication (i.e., mixing the medication prior to the injection), waiting during medication preparation, and needle handling. Every participant valued all seven health states.

**Results:**

A total of 209 participants completed interviews (57.4% male; mean age = 60.4y). The mean utility of the oral treatment health state was 0.89. All injection health states had significantly (*p* < 0.01) lower utilities ranging from 0.86 to 0.88. Differences among health state utilities suggest that each administration requirement had a small but measureable disutility: -0.004 (reconstitution), -0.004 (needle handling), -0.010 (reconstitution, needle handling), and -0.020 (reconstitution, waiting, needle handling).

**Conclusions:**

Findings suggest it is feasible to use the TTO method to quantify preferences among injection treatment processes. It may be useful to incorporate these utility differences into cost-utility models comparing weekly injectable treatments for patients with type 2 diabetes.

## Background

Cost-utility analyses (CUAs) are often conducted to estimate the value of treatments for type 2 diabetes, and results are used to inform decisions on allocation of healthcare resources [1–3]. In CUAs, treatments are assessed in terms of cost per quality-adjusted life year (QALY) gained [4]. QALYs are calculated with health state utilities, which are values representing the strength of preference for a given health state [5, 6]. For type 2 diabetes, a range of utilities have been published, including utilities derived from generic preference-based measures administered to large samples of patients [7–13]. These utilities are useful for representing treatments that vary in clinical outcomes such as glycemic control and diabetes symptom severity. However, treatments for type 2 diabetes vary not only in treatment effectiveness, but also in treatment process attributes such as route of administration and dose frequency [14, 15].

There is growing awareness that the treatment process can impact patients’ quality of life, treatment preference, and treatment adherence [16–20]. Therefore, recent research has aimed to quantify the impact of treatment process attributes on utility so that these values can be incorporated into CUAs [21]. Treatments for type 2 diabetes, in particular, vary substantially in terms of the treatment process, and two studies have examined the impact of treatment process attributes including injection frequency and dose flexibility on utility in the context of type 2 diabetes [22, 23]. In addition, the diabetes research and regulatory communities have recently emphasized the assessment of factors beyond HbA1c that are likely to be important to patients, such as treatment process attributes [24]. These treatment process variables are most likely to influence the outcome of cost-utility modeling when comparing treatments that are similar in terms of efficacy and safety, which is the case for some of the glucagon-like peptide-1 (GLP-1) receptor agonists.

GLP-1 receptor agonists are a class of injectable treatments for patients with type 2 diabetes that is often recommended as part of combination therapy when oral medication does not result in sufficient glycemic control [15, 25–27]. Several GLP-1 receptor agonists are now available as weekly injections [28–30]. These treatments have some important strengths and weaknesses in common, including effectiveness for lowering HbA1c, fasting blood glucose, and body weight [25, 31, 32]; low risk of hypoglycemia [32]; and gastrointestinal adverse events [26, 31, 32]. Despite these commonalities, these weekly treatments and their injection devices differ in terms of treatment process attributes, such as needle handling and steps required to prepare the medication. Although differences could have an impact on patients’ preferences, no utility values are available to represent these attributes of weekly GLP-1 receptor agonists.

Therefore, the purpose of this study was to estimate utilities associated with treatment process attributes of weekly GLP-1 receptor agonists. This study focused on the disutility that may be associated with three treatment-related attributes that distinguish among these medications: (1) requirements for reconstitution (i.e., mixing the medication) prior to injection, (2) required waiting time prior to the injection, and (3) the need to handle the injection needle. The first two attributes, reconstitution and waiting, were hypothesized to be important to patients who preferred to minimize complexity and inconvenience in their treatment regimens.

The third attribute, needle handling, is considered to be an important safety issue. The “Health and Safety (Sharp Instruments in Healthcare) Regulations 2013” were issued in the UK to provide guidance for employers and employees [33]. This guidance aims to reduce risks of medical sharps injuries by implementing aspects of the European Council Directive 2010/32/EU, often referred to as the “Sharps Directive” [34]. The 2013 regulations recommend using “safer sharps,” which are medical sharps that minimize risk of accidental injury, such as injection devices that do not require needle handling or include mechanisms to cover the needle after use. This injection device attribute, which varies among weekly GLP-1 receptor agonists, was hypothesized to have an impact on patient preference and utility.

## Methods

### Overview of study design

Because generic preference-based measures such as the EQ-5D do not specifically assess the impact of treatment process or treatment convenience, generic instruments are unlikely to be sensitive to differences related to treatment process attributes. Therefore, utilities were estimated using vignette-based methods, which are well-suited for isolating the impact of specific treatment-related attributes on utility. These methods were similar to those of two previously published studies that estimated the utility impact of treatment-related attributes in the context of type 2 diabetes [22, 35]. The utilities derived in these previous studies have been used in a range of published CUAs of treatments for type 2 diabetes [36–39]. The health states in the current study used the same basic description of type 2 diabetes that had been used in these two previous utility studies, with additional content describing weekly injectable treatment and variations in the injection devices.

Like the two previous studies, the current study estimated utilities for treatment-related attributes based on a valuation study in a sample of patients with type 2 diabetes. First, health state vignettes were developed and refined based on clinician interviews, published literature, injection device instructions for use, and a pilot study. Then, the health states were valued by patients in a time trade-off (TTO) task with a 20-year time horizon. By comparing utility values across the health states, it was possible to estimate the disutility associated with various combinations of treatment process attributes.

### Health state development

Health state descriptions (often called vignettes or scenarios) were drafted based on health states administered in two previous studies [22, 35], published literature [40–42], interviews with four clinicians who had research and clinical experience with type 2 diabetes (all with MD degrees), and instructions for use of the three devices associated with weekly GLP-1 receptor agonists [28–30]. First, health states administered in the two previous studies were adapted for the description of type 2 diabetes in all seven health states. Then, a literature search and clinician interviews were conducted to identify injection device attributes that were likely to be important to patients and differentiate among devices used to inject weekly GLP-1 receptor agonists. In addition, descriptions provided by clinicians were used to inform the development of health state content. Finally, instructions for use of the three available GLP-1 receptor agonist devices were used to finalize details of health state content, including the images and descriptions presented on the device display page (described below).

All health states were presented on individual cards, each with a series of bullet point descriptions. Health state text is presented in the paragraphs below. To ensure that respondents had a clear understanding of the health states, a device display page was given to each participant. This page summarized and illustrated characteristics of an injection pen and the three injection process attributes. The descriptions were derived from the instructions for use of each injection pen (Appendix A), and the illustrations were taken directly from these instructions [28–30]. Prior to presenting health states, the interviewer reviewed this device display page with each participant to ensure that he/she/they understood the relevant concepts. Respondents also referred to the device display page as needed throughout the interview.

The first health state (health state A), called the basic health state, described a patient with type 2 diabetes on oral treatment: “You have had type 2 diabetes for several years. You are at your current weight. You take oral medication (pills or tablets). Your blood sugar levels are usually in control, but sometimes your blood sugar is too high or too low. If your blood sugar level is too low, you may experience dizziness/light-headedness, sweating, or shaking. If your blood sugar level is too high, you may experience tiredness, blurred vision, thirst, or frequent urination.” This health state, which provided context for the injection-related attributes described in subsequent health states, was based on health states administered in two previous studies [22, 35].

Six additional health states (i.e., health states B to G or “the injectable health states”) started with the same content as health state A, but added a weekly injectable treatment. After the descriptions from health state A, each of these six health states included the same bullet point: “In addition to taking oral medication (pills or tablets), you give yourself an injection ONCE EACH WEEK using a device called an injection pen.” Then, these health states included three statements, each representing an injection process attribute.

The three injection process attributes (reconstitution, waiting, and needle handling) were selected because they were likely to differentiate among the three currently available once-weekly GLP-1 receptor agonists: albiglutide [30], dulaglutide [29], and once-weekly exenatide [28]. The first attribute was the requirement for reconstitution, referred to as “mixing.” Albiglutide and once-weekly exenatide both require reconstitution of the medication prior to the injection. It was hypothesized that some participants would prefer health states without this requirement. The description of reconstitution on the device display page was designed to be general enough to apply to both albiglutide (which requires “rocking” the pen) and once-weekly exenatide (which requires “tapping” the pen). Each of the injection health states included one of the following two descriptions: “You follow a sequence of several steps to mix the medication before injecting” or “When you open the package, the medication is ready to be injected. You do NOT need to mix the medication prior to injection.”

The second attribute was called “waiting.” As part of the reconstitution process, albiglutide requires a waiting period of 15 to 30 minutes, while neither of the other two weekly treatments require waiting. It was hypothesized that some participants would prefer not to have this extra requirement. The waiting requirement was represented by one of the following two statements: “During the steps required for mixing the medication, you have to stop and wait 15 to 30 minutes for the medication to mix” or “During the steps required for mixing the medication, you do NOT have to stop and wait for the medication to mix.”

The third attribute was the requirement to handle the needle. Whereas albiglutide and once-weekly exenatide require the patient to attach a needle to the injection pen for each injection, the dulaglutide pen includes a pre-attached needle that automatically retracts into the device after use. It was hypothesized that some respondents may have a preference for an injection device that does not require needle handling. Each of the injection health states included one of the following two descriptions: “You have to handle the needle and attach it to the pen” or “When you open the injection pen package, the needle is already included as a part of the injection pen. You do NOT need to handle the needle or attach it to the pen.”

### Participants

Participants were required to meet these inclusion criteria: (1) diagnosed with type 2 diabetes by a recognized medical professional; (2) be between the ages of 30 and 75 years old; (3) able to identify the age at which they were first diagnosed with diabetes; (4) able to read and understand English; (5) willing and able to give informed consent. Participants were not eligible if they had a cognitive impairment, hearing difficulty, or severe pathology that could interfere with their ability to complete the interview. To verify diagnosis of type 2 diabetes, participants receiving pharmaceutical treatment were required to bring proof of medication to the interviews (e.g., medication packaging or a letter from a doctor). Participants who were not taking medication were required to describe their symptom history, diagnosis process, and disease management strategies at a level of detail suggesting that they were honestly reporting their diagnoses.

Participants were recruited via newspaper advertisements, online advertisements, fliers posted near interview sites, and advertisements in newsletters distributed by patient advocacy groups. A total of 301 individuals were screened to assess whether they met inclusion criteria. Of the 301 screened participants, 44 were ineligible because they did not meet inclusion criteria. Of the 257 who were eligible, 235 were scheduled, and 214 attended their interviews. Three of the 214 participants were unable to sufficiently understand the utility interview procedures in order to provide valid data, and two participants were determined to be ineligible upon rescreening prior to consent (one was unable to complete protocol requirements due to visual impairment, and the other was unable to provide proof of treatment or diagnosis). Thus, a total of 209 (150 Scotland, 59 England) valid interviews were completed.

### Pilot study

To assess the comprehensibility of the draft health states and determine whether the TTO methods were feasible for valuing these particular health states, a pilot study was conducted with 26 patients with type 2 diabetes in London, United Kingdom (UK) (84.6% male; mean age = 57.7 years; age range = 35 to 73). Participants valued the health states using TTO methods with 10-year and 20-year time horizons. The TTO method with both time horizons was easy for participants to understand and complete. The 20-year time horizon was selected over 10 years because it better represents the chronic nature of type 2 diabetes.

During the pilot study, the health states were revised based on patient feedback in order to maximize clarity and comprehension. To shorten health states, some of the details about injection pens and the treatment process attributes were deleted from the health states and presented instead on the device display page. This change appeared to make the task easier for participants. After finalizing the health states and methods, participants consistently reported that the health states, device display page, and TTO procedures were clear and easy to understand.

### Utility interview procedures and scoring

After the pilot study, health state utilities were elicited in a TTO valuation study in four UK locations: London, England, and three locations in Scotland (Inverness, Portree, and Edinburgh). All participants provided written informed consent. Procedures and materials (for both the pilot study and the full utility valuation study) were approved by an independent Institutional Review Board (Ethical & Independent Review Services; Study Number 15022). In each geographic location, interviews were conducted at a facility with multiple private offices so that every one-on-one interview could be conducted in a quiet private room. There were five interviewers (including three of the study authors and two additional interviewers mentioned in the acknowledgments section). The utility assessment was conducted by following a semi-structured interview script in order to standardize the utility assessment procedures. The interview team was trained by the principal investigator, who also observed each interviewer multiple times to ensure that procedures were followed consistently.

In each interview, the basic health state (health state A) was presented first. Then, the device display page was presented, followed by the six health states describing treatment with injectable medication (B to G) in random order. Every participant valued all seven health states. During this process, interviewers reviewed the treatment process attributes using the standardized language on the device display page. As an introductory task, participants were asked to rank the health states in order of preference. Then, participants valued the health states in a TTO task with a 20-year time horizon and 1-year (i.e., 5%) trading increments. Following commonly used procedures for each health state [4], participants were offered a choice between spending 20 years in the health state versus spending varying amounts of time in full health. Choices alternated between longer and shorter time periods (i.e., 20 years, 0 years [i.e., dead], 19 years, 1 year, 18 years, 2 years, 17 years, 3 years…). For each health state, the utility value was calculated based on the point of indifference between *y* years in the health state being evaluated (i.e., 20 years) and *x* years in full health (followed by dead). The resulting utility estimate (*u*) was calculated as *u = x/y*. For example, if 20 years in a health state was perceived to be equally preferable to 16 years in full health, the utility of that health state would be 0.80 (i.e., 16/20).

If a participant indicated that a health state was worse than dead, the task and scoring procedures were altered as described in previous literature [43, 44]. Participants were offered a choice between dead (choice 1) and 20 years (choice 2) beginning with varying amounts of time in the health state being rated, followed by full health. Then, utility scores were calculated with a bounded scoring approach (*u = −x / t*, where *x* is the time in full health, and *t* is the total life span of choice 2), which is commonly used to avoid highly skewed distributions for negative utility scores.

### EQ-5D-3L and EQ-5D-5L

The EQ-5D-3L and EQ-5D-5L were administered to characterize the sample in terms of quality of life and overall health status. Both versions were administered to all participants because there is interest in comparing results of the newer EQ-5D-5L with results from the more established EQ-5D-3L [45].

The EQ-5D is a self-administered, generic, preference weighted measure designed to assess health status [46–48]. The original version with three levels per domain (EQ-5D-3L) and the newer version with five levels per domain (EQ-5D-5L) were both administered. In each version, the first section consists of five dimensions to assess HRQL (mobility, self-care, usual activities, pain/discomfort, and anxiety/depression). These five dimensions are scored based on preference weightings to obtain an “index score” that is often used as a utility in economic modeling. The second section consists of a visual analogue scale on which respondents rate their current health, with anchors of 0 (“worst imaginable health state”) and 100 (“best imaginable health state”). Half the participants were randomized to complete the EQ-5D-3L first, while the other half completed the EQ-5D-5L first. The EQ-5D-3L index score was computed using the UK “MVH” tariffs [49], and the EQ-5D-5L index score was computed with the recently published value set derived from a general population sample in England [50].

### Statistical analysis procedures

Statistical analyses were completed using SAS version 9.4 (SAS Institute, Cary, NC). Continuous variables were summarized in terms of means and standard deviations. Categorical demographic variables were summarized as frequencies and percentages. Demographic subgroups were compared with chi-square analyses (for categorical variables) and t-tests (for continuous variables). Pairwise comparisons between health state utilities were performed using t-tests. Disutilities (i.e., decreases or differences in utility) of injection process attributes were calculated by subtracting the mean utility of one health state from another.

## Results

### Sample description

A total of 209 participants with type 2 diabetes completed the utility interview, including 150 in Scotland and 59 in England (see demographics in Table 1). Current treatment regimens for type 2 diabetes included oral medication (*n* = 139; 66.51%), injectable medication (*n* = 10; 4.78%), combined oral and injectable treatment regimens (*n* = 36; 17.22%), and no medication (*n* = 24; 11.48%). All 185 participants receiving pharmaceutical treatment brought proof of medication to the interviews. The 24 participants who were not taking medication described their symptom history, diagnosis process, and disease management at a level of detail strongly suggesting that they were honestly reporting their diagnoses. The most commonly reported comorbid health conditions were hypertension (34%), depression (25%), arthritis (25%), anxiety (16%), angina (10%), diabetic retinopathy (9%), and heart attack or heart disease (8%).

**Table 1:** Demographic characteristics

Characteristics	Scotland (*N* = 150)	England (*N* = 59)	Participants in the Analysis Sample (*N* = 209)	*P*-value^a^
Age (Mean, SD)	61.2 (9.3)	58.2 (7.5)	60.4 (8.9)	0.027
Gender (n, %)				
Male	85 (56.7%)	35 (59.3%)	120 (57.4%)	0.73
Female	65 (43.3%)	24 (40.7%)	89 (42.6%)	
Ethnicity (n, %)				
White	150 (100.0%)	31 (52.5%)	181 (86.6%)	<0.0001
Mixed	0 (0.0%)	5 (8.5%)	5 (2.4%)	
Asian	0 (0.0%)	8 (13.6%)	8 (3.8%)	
Black	0 (0.0%)	15 (25.4%)	15 (7.2%)	
Marital Status (n, %)				
Single	15 (10.0%)	18 (31.0%)	33 (15.9%)	0.0006
Married/Living with partner	105 (70.0%)	28 (48.3%)	133 (63.9%)	
Other^b^	30 (20.0%)	12 (20.7%)	42 (20.2%)	
Employment Status (n, %)				
Full-time work	23 (15.3%)	13 (22.0%)	36 (17.2%)	0.051
Part-time work	24 (16.0%)	16 (27.1%)	40 (19.1%)	
Other^c^	103 (68.7%)	30 (50.8%)	133 (63.6%)	
Education Level (n, %)				
University degree	40 (26.7%)	24 (40.7%)	64 (30.6%)	0.048
No University degree	110 (73.3%)	35 (59.3%)	145 (69.4%)	

^a^ *P*-values are based on t-tests for continuous variables and chi-square analyses for categorical variables

^b^Other marital status includes divorced (*n* = 19), separated (*n* = 8), and widowed (*n* = 15)

^c^Other employment status includes homemaker/housewife (*n* = 1), student (*n* = 4), unemployed (*n* = 13), retired (*n* = 91), disabled (*n* = 22), carer (*n* = 1), and transient (*n* = 1)

*SD* standard deviation

**Table 2:** EQ-5D scores

Measure	N	Mean	SD	Range
ED-5D-3L^a^				
Index Score	206^b^	0.76	0.27	-0.08–1.00
VAS	207^c^	75.51	16.29	25.00–100.0
EQ-5D-5L^a^				
Index Score	209	0.83	0.21	0.02–1.00
VAS	209	76.13	16.57	15.00–100.0

^a^Higher scores for the five individual items indicate greater problems. Higher scores for the index score and the VAS indicate greater utility and quality of life

^b^Two participants did not provide a response to the anxiety/depression item, and one participant provided two responses to the pain/discomfort item

^c^Two participants did not complete the VAS

*SD* standard deviation, *VAS* visual analogue scale, *UK* United Kingdom

**Table 3:** Utilities and disutilities

Health States	Mean Utility^a^	SD	Disutilities: Difference from Health State A		Disutilities: Difference from Health State G	
			Mean	SD	Mean	SD
A. Basic health state (Oral only)	0.888	0.120	–	–	–	–
Health States with Oral and Injectable Treatment^b^						
B. Reconstitution, waiting, needle handling	0.858	0.165	-0.030	0.073	-0.020	0.042
C. Reconstitution, waiting	0.863	0.161	-0.025	0.066	-0.014	0.032
D. Reconstitution, needle handling	0.868	0.159	-0.020	0.063	-0.010	0.027
E. Reconstitution	0.874	0.157	-0.014	0.058	-0.004	0.016
F. Needle handling	0.874	0.156	-0.014	0.058	-0.004	0.015
G. No inconveniences	0.878	0.156	-0.010	0.056	–	–

^a^TTO scores are on a scale anchored with 0 representing dead and 1 representing full health

^b^Health states B to G include the basic health state, plus treatment with a weekly injection

*SD* standard deviation

The EQ-5D-3L and EQ-5D-5L mean index were scores similar to those published previously for patients with type 2 diabetes without complications [51]. The score ranges and standard deviations suggest that the sample was diverse in terms of health status (Table 2).

### Health state utilities

In the introductory task, respondents generally ranked health states in a logical order. Health states with more treatment administration steps were typically ranked as less preferable, with rankings ranging from 1 (most preferable health state) to 7 (least preferable health state). The oral-only health state (A) had a mean ranking of 1.08, followed by G (2.01), E (3.54), F (3.59), D (5.24), C (5.57), and B (6.98).

The mean utility scores followed the same order as the health state rankings, with greater numbers of treatment administration steps associated with lower utility (Table 3). The mean utility for health state A describing diabetes with oral treatment was 0.888, while adding the weekly injection in health state G reduced utility to 0.878. Each additional inconvenience associated with the weekly injection decreased utility further, and the lowest utility value was for health state B, which described reconstitution, waiting, and needle handling (0.858). T-tests found no statistically significant differences in utility between men and women; between older and younger respondents (categorized by median split); or between respondents from England and Scotland.

All but one participant rated every health state as better than dead (i.e., utility score > 0). One participant rated every health state as worse than dead. When asked to explain these TTO choices, the participant explained that she had negative experiences with oral medications in the past, and therefore she strongly preferred not to live in a health state requiring oral medication.

### Disutilities and comparisons among health states

Disutility scores (i.e., decreases in utility) were calculated relative to both oral-only treatment as described in health state A and oral plus weekly injection treatment as described in health state G (Table 3). Disutilities for the three injection process attributes (i.e., reconstitution, waiting, needle handling) can be obtained based on the difference between health state G and health states B to F. For example, health states G and F are identical except for the needle handling requirement in F. Therefore, the difference between G and F (-0.004) represents the disutility of the needle handling requirement in the context of a weekly injection for treatment of type 2 diabetes. Disutilities of individual injection attributes were of a relatively small magnitude, such as the disutility of the requirement for reconstitution (-0.004). However, health states representing combinations of multiple injection attributes had larger disutilities, including a disutility of -0.020 for health state B representing the combination of reconstitution, waiting, and needle handling requirements.

T-tests were conducted to explore whether differences among health state utilities were statistically significant. The utility of the oral-only health state (health state A) was found to be significantly different from all other utilities (*p* < 0.0001 for health state A vs B, C, D; *p* = 0.0007 vs. E and F; *p* = 0.0098 vs. G). The injection health state without any of the three inconveniences (health state G) was compared to each of the other injection health states (B to F). The utility of G was found to be significantly different from utilities of all health states that included reconstitution, waiting, and/or mixing (*p* < 0.0001 vs. B, C, D; *p* = 0.0003 vs. F; *p* = 0.0007 vs E).

## Discussion

The results of this study add to previously published utilities associated with attributes of treatment for type 2 diabetes. Three prior studies have reported that patient preference and corresponding utility scores are affected by a wide range of treatment attributes, including efficacy, adverse events, effects on body weight, dose frequency, and dose flexibility [22, 23, 35]. The current study extends this research by suggesting that the injection treatment process may also have an impact on utility. Each individual injection inconvenience examined in this study (i.e., reconstitution, waiting, needle handling) had a small impact on utility when presented on its own. However, simultaneous multiple inconveniences, such as health state C combining reconstitution with waiting time, had a more substantial impact on utility. These results are consistent with previous studies in which patients with type 2 diabetes reported preferences among available injection devices, largely influenced by each device’s ease-of-use [52–57].

Taken together, current and previous results indicate that treatment process attributes such as dose frequency and injection convenience have an impact on preferences of patients with type 2 diabetes. Furthermore, it is feasible to quantify these preferences in terms of utilities that may be used in cost-utility models examining and comparing the value of available treatments. In the current study, the disutilities associated with inconveniences of injection delivery systems range from -0.004 for a single inconvenience to -0.020 for a combination of three inconveniences. The magnitudes of these utility differences are similar to those previously reported for other treatment process variables, such as route of administration and other injection attributes, which typically range from 0.01 to 0.05 [21, 22, 43]. In general, treatment process utility differences are smaller than differences that would be expected for health states differing in clinical outcomes or disease severity. However, even small differences in utility can have a meaningful impact on the outcome of a cost-utility model, particularly when modeling many patients with a chronic disease over an extended time period. Therefore, the current utility values may be useful in cost-utility models comparing weekly GLP-1 receptor agonists for treatment of type 2 diabetes.

When using these values, modelers should focus on differences among utilities of health states B through G, which are presented in Table 3. These six health states are identical to each other except for differences in the three injection-related attributes (i.e., reconstitution, waiting, needle handling). Therefore, any differences in utility can be attributed specifically to these three attributes. These utility differences may be used in models comparing weekly GLP-1 receptor agonists that differ in the three injection attributes. For example, a model could compare a medication requiring reconstitution, waiting, and needle handling to a medication administered without these inconveniences. For this model, the utility difference between health states B and G would be relevant, and the corresponding disutility of -0.020 may be applied to the treatment arm representing the injection pen with the three inconveniences.

Although current results are potentially useful, the vignette-based method used in this study is not standardized, and comparability to utilities derived via other methods is uncertain. Some health technology assessment (HTA) guidelines, such as the guide issued by NICE, indicate a preference for utilities derived from generic preference-based measures such as the EQ-5D to maximize “consistency across appraisals” [58]. However, generic measures are not sensitive to all aspects of disease and treatment that may be important for patients, and therefore, HTA guidelines allow for methodological flexibility. For example, the NICE guide (2013) says utilities estimated using other methods may be acceptable when the EQ-5D is not “appropriate” [58]. One situation when generic measures are likely to be inappropriate is the assessment of utilities representing preferences for treatment process attributes, such as injection attributes. Generic instruments such as the EQ-5D assess broader domains of health, rather than specific treatment attributes. In contrast, vignette-based methods are well-suited for isolating the utility impact of the treatment process because vignettes can be designed to vary specific treatment process attributes while disease severity and treatment outcome are held constant. A recent systematic review found that 14 of 15 *process utility* studies used vignette-based methods, suggesting consensus that this methodology may be the best strategy for estimating utilities of treatment process attributes [21].

Still, the resulting utility scores should be interpreted and used with caution because vignette-based utilities could be subject to biases. For example, scores could be affected by a focusing effect, leading respondents to attend closely to small differences among health states. Furthermore, the accuracy of utilities is limited by the content and level of detail in the health state descriptions, which cannot capture the totality of the patient experience. Despite these limitations, the current vignette-based utilities reflect genuine patient preferences for attributes of currently available weekly GLP-1 receptor agonists. Therefore, by using these utility values in cost-utility analyses, modelers may be able to more accurately represent differences among these treatments.

Vignette-based studies also raise the issue of whether the disease should be named as part of the health state description. Some studies have suggested that the disease label can influence the resulting utility scores, while other studies have reported situations when the label did not affect results [59–61]. Some researchers recommend omitting the disease label to avoid risk of bias, but others include the label to maximize health state clarity. The health state descriptions in this study included the label (i.e., type 2 diabetes) because an unambiguous presentation of the disease was necessary to provide context for the injection process. Furthermore, because the study was conducted in a sample of patients with type 2 diabetes, the disease described in the health states would likely have been obvious to the respondents, even without the label. Therefore, it may have been perceived as odd or confusing if the disease were not named. In addition, the disease label is unlikely to interfere with the goal of this study, which was to estimate utility differences among injection devices rather than to estimate the utility of type 2 diabetes.

Another issue is whether health states should be valued by patients or general population respondents. Both types of samples have strengths and limitations. Some HTA agencies prefer that utilities represent the general population or societal perspective so that these broader values are represented in models informing healthcare resource allocation [58, 62, 63]. In the current study, a patient sample was used for two reasons. First, patients are likely to have a greater understanding of the disease, treatment, and ongoing impact of injection device convenience. Second, three previous studies estimating utilities of attributes of type 2 diabetes treatments have been conducted in patient samples [22, 23, 35], and the current study was designed to methods guides: guide to the methods of technology appraisal be comparable to those studies in order to maximize consistency when utilities from multiple studies are used in a single model. However, in HTA appraisals where general population perspective is preferred, the patient sample would be considered a study limitation.

## Conclusions

This study found that three attributes of weekly injection devices had a measurable impact on patient preference. The resulting utility values may be useful for differentiating among weekly GLP-1 receptor agonists in cost-utility modeling used to inform decisions about healthcare resource allocation. Furthermore, findings suggest that injection device attributes may be important to some patients, and therefore, it may be useful for clinicians to consider these device attributes when choosing medication for patients initiating these weekly treatments.

## Appendix A. Text from the Device Display Page

### Injection Pen

- You give yourself an injection **ONCE EACH WEEK** using a device called an injection pen.
- The device looks similar to a pen that you would use for writing. It is a little larger than a typical writing pen.
- Each pen is only used once, and each pen comes filled with a single dose of medication.

### Mixing Medication

- You follow a sequence of **several steps to mix the medication** before injecting.
- The pen contains liquid and powder in separate compartments.
- The following steps must be completed:
  - **– Twist** a part of the device to combine the liquid and powder.
  - – Mix the medication to ensure that the powder is fully dissolved in the liquid. You can mix the medication by either **rocking** the device back and forth (like a windscreen wiper) or by **tapping** the device against the palm of your hand several times.
  - – By looking through a small clear window in the pen, you can see the mixed medication in the pen. **Look at the mixed medication** to ensure that the powder is fully dissolved. If the powder is not fully dissolved, further mixing is needed.
- After these steps are completed, the medication is ready to be injected.

### Waiting Between Steps for Mixing Medication

- During the steps required for mixing the medication, you have to **stop and wait 15 to 30 minutes** for the medication to mix.
- This waiting time is in addition to the time required for the steps you take to mix the medication.)

### Handling and Attaching the Needle

- The needle is provided in the same package as the injection pen, but the **needle is separate** from the pen when you open the package.
- You have to **handle the needle** and attach it to the pen.

## Abbreviations

CUA: Cost-utility analysis
GLP-1: Glucagon-like peptide-1
HbA1c: Glycated hemoglobin
HRQL: Health-related quality of life
HTA: Health technology assessment
MVH: Measurement and Valuation of Health
NICE: National Institute for Health and Care Excellence
PRO: Patient-reported outcomes
QALY: Quality of life year
TTO: Time trade-off
UK: United Kingdom
VAS: Visual analogue scale
